# Characterization of uniquely tumorigenic cancer stem cells in salivary gland adenoid cystic carcinoma

**DOI:** 10.3389/froh.2025.1570042

**Published:** 2025-04-30

**Authors:** Kristy A. Warner, Sosuke Sahara, Alexandra E. Herzog, Felipe Nör, Rogerio M. Castilho, Peter J. Polverini, Jacques E. Nör

**Affiliations:** ^1^ Department of Cariology, Restorative Sciences, Endodontics, University of Michigan School of Dentistry, Ann Arbor, MI, United States; ^2^ Department of Periodontics and Oral Medicine, School of Dentistry, Ann Arbor, MI, United States; ^3^ Department of Otolaryngology, University of Michigan School of Medicine, Ann Arbor, MI, United States; ^4^ Rogel Cancer Center, University of Michigan, Ann Arbor, MI, United States

**Keywords:** salivary gland cancer, cancer stemness, tumor-initiating cells, aldehyde dehydrogenase, tumor cell heterogeneity

## Abstract

**Background:**

Cancer stem cells (CSC) are endowed with multipotency, self-renewal and unique tumorigenic potential. They have been identified as cells with high activity of aldehyde dehydrogenase (ALDH) and high expression of CD44 in head and neck squamous cell carcinoma (HNSCC). The objective of this work is to understand whether salivary gland adenoid cystic carcinoma contain CSCs and whether they exhibit a unique tumorigenic activity in this cancer.

**Methods:**

We used flow cytometry, salisphere and western blot assays with 3 human ACC cell lines (UM-HACC-2A, UM-HACC-14, UM-HACC-6) to characterize the impact of ALDH activity and CD44 expression. *In vitro* results were verified *in vivo* by orthotopic injection of cells retrieved from a patient-derived xenograft (PDX) model of ACC (UM-PDX-HACC-14) in the submandibular gland of SCID mice. Primary tumor and metastatic spread were evaluated by 2 pathologists blinded for experimental conditions.

**Results:**

The fraction of ALDH^high^CD44^high^ cells in UM-HACC-2A, UM-HACC-14 and UM-HACC-6 ranged from 3% to 8%. ALDH^high^CD44^high^ cells formed more salispheres and expressed higher levels of stem cell markers (e.g., Notch2, Bmi-1) compared to control ALDH^low^CD44^low^ cells. ALDH^high^CD44^high^ cells sorted from the ACC PDX tumors were more tumorigenic upon orthotopic transplantation into submandibular salivary glands and generated more lung metastases than control ACC cells. Strong ALDH1A1 staining was observed in the majority of salivary gland tumors and lung metastases generated by transplantation of ALDH^high^CD44^high^ cells.

**Conclusions:**

We conclude that salivary gland adenoid cystic carcinoma contain a small population of uniquely tumorigenic cells characterized by high ALDH activity and expression.

## Introduction

There is a major need for a safe and effective therapy for patients with malignant salivary gland cancer. Adenoid cystic carcinoma (ACC) is a rare, slow-growing, cancer that is characterized by poor long-term outcome for patients (1, 2). ACC tumors are highly variable, consist of multiple cell types, arise from different primary sites, and tend to occur more frequently in women than men (1–5). In addition, patients with ACC tumors have diverse demographics (age, ethnicity, location of primary tumor), and have complex cases (grade, stage, +/−peri-neural, hematogenic invasion) making it difficult to identify effective treatments (1–7). The standard of care for ACC patients is surgery and radiation, with no effective and safe systemic therapies (5, 8–11). Identifying a “universal target” such as cancer stem cells, constitute an attractive strategy to overcome the intrinsic challenges associated with the slow, albeit relentless, pattern of progression exhibited by ACCs. Here, we investigated whether CSCs play a functional role in the pathobiology of ACC.

Cancer stem cells (CSC) are a rare and unique cell population found in many different solid tumors, including breast, glioblastoma, head and neck, prostate, lung, colon, pancreatic and liver cancer (12). CSC proliferate relatively slowly, are endowed with self-renewal, are multipotent, highly tumorigenic, express high levels of Bmi-1 and contribute to metastases, recurrence, and to chemoresistance (12, 13). Notably, Bmi-1 is a master regulator of self-renewal and stemness in both, physiological and malignant cells (13).

Cancer stem cells were first identified in solid tumors of breast cancer (14). Al-Hajj and colleagues identified CSCs as CD44 ^+^ CD24^low^ in breast cancer (14). This tumorigenic cell population increased at very low numbers and generated phenotypically diverse tumors *in vivo*. ALDH1^high^ cells also identified CSC in human breast cancer and head and neck tumors (15, 16). A few years later, Prince and colleagues identified CD44 + cells as CSCs in HNSCC tumors (17). CD44 is a transmembrane glycoprotein involved in cell survival, motility, and differentiation as a CSC marker in head and neck squamous cell carcinomas (18). The CD44 + cell population combined with ALDH^high^ expression resulted in phenotypically diverse head and neck squamous cell carcinoma tumors upon serial dilution *in vivo* (19–22).

Adams and colleagues hypothesized that salivary gland CSCs might be responsible for treatment failure in these patients (23). Recent studies in head and neck squamous cell cancer and salivary gland mucoepidermoid carcinoma have shown ALDH^high^CD44^high^ cells exhibit characteristics consistent with CSCs and are resistant to platinum-based chemotherapy (24–26). Targeting HNSCC or mucoepidermoid carcinoma CSCs with IL-6R inhibitor (tocilizumab), small molecule inhibitors of MDM2-p53 interaction (MI-773, APG115), mTOR inhibitors (rapamycin, temsirolimus) reduced the CSC fraction, sensitized tumors to chemotherapy and inhibited tumor progression (26–30). Similarly, single agents such as small-molecule inhibitors of Bcl-2 or MDM2 inhibited ACC tumor growth *in vivo* (31, 32). Notably, a combination therapy using MI-773 and cisplatin significantly reduced CSC fraction, diminished tumor growth rates and prevented ACC recurrence for more than 300 days in mice (33). And finally, Sahara and colleagues reported significant reductions in CSC fraction and tumor recurrence using combination therapy of cisplatin and a small molecule inhibitor of a Bmi-1 (PTC-596, Unesbulin) *in vivo* (34).

Collectively, these studies suggest that targeting ACC CSCs might be beneficial for patients. A direct connection of ALDH^high^CD44^high^ expression to ACC CSC phenotype has been less clear. While some reports showed that human ACC ALDH^high^ cells were more tumorigenic (35, 36), others reported no correlation between ALDH1 tumor cell expression and perineural invasion, patient survival in ACC or other clinical parameters (37). In addition, Notch and Nanog have also been implicated in salivary gland cancer stemness (38–40). Here, we used 3 unique human ACC cell lines *in vitro,* and a matching UM-PDX-HACC-14 model *in vivo* (41, 42) to test the hypothesis that ALDH^high^CD44^high^ cells function as uniquely tumorigenic CSCs in salivary gland adenoid cystic carcinoma.

## Materials and methods

### Cell culture, western analysis, STR profiling

Human salivary gland ACC cells (UM-HACC-2A, UM-HACC-14, UM-HACC-6) were cultured in Salivary Gland Medium (SGM) consisting of Dulbecco's Modified Eagle's Medium (DMEM) (Invitrogen, Wlatham, MA), supplemented with 1% L-glutamax (Invitrogen), 1% AAA antibiotic (Sigma-Aldrich), 1% Amphotericin B (Sigma-Aldrich), 10% Fetal Bovine Serum (FBS; R and D Systems), 20 ng/ml rhEGF (R&D Systems), 0.4 mg/ml human hydrocortisone (StemCell Technologies, Vancouver, Canada), 5 µg/ml human insulin (Sigma-Aldrich, St. Louis, MO) (25, 27, 34, 41, 42).

Proteins were extracted using NP-40 lysis buffer (24–34). Lysates were collected from cells grown in attached or ultra-low attachment (ULA) conditions (salispheres) or from ACC cells sorted for ALDH activity (Aldefluor; StemCell Technologies) and/or CD44-APC (BD Pharmingen) expression. Proteins were resolved on SDS-PAGE gels and membranes were probed using antibodies from Santa Cruz Biotechnology (Santa Cruz, CA, USA), as follows: ALDH1/2 (SC-166362), ALDH1A1 (SC-374149), β-actin (SC-4778); and from Cell Signaling (Cell Signaling Technology, Danvers, MA), as follows:, STAT3 (30385), p-STAT3 (9138), Bmi-1 (6964), Nanog (4903), NOTCH1 (3608), NOTCH2 (4530). The identity and purity of ACC cells were routinely confirmed via STR profiling, (Genetica, Burlington, NC) (32, 41, 42).

### Flow cytometry, orthotopic ACC model

For CSC analysis (2–4 × 10^5^ UM-HACC-2A, UM-HACC-14, UM-HACC-6) or sorting (20–30 × 10^6^) cells were preincubated with 10 µl of the ALDH inhibitor diethylaminobenzaldehyde (DEAB) for 10 min at 37°C, directly stained with Aldefluor (StemCell Technologies) for 30 min, CD44-APC (BD Pharmingen, Franklin Lanes, NJ, USA) for 10 min at 4°C, and DAPI (cell viability marker, Invitrogen) as previously described (25–31, 33, 34). ALDH^low^CD44^low^, ALDH^low^CD44^high^, ALDH^high^CD44^low^ and ALDH^high^CD44^high^ groups were analyzed or collected for experiments. For *in vivo* serial dilution experiments, UM-PDX-HACC-14 tumors were dissociated in collagenase-hylauronidase (StemCell Technologies), as previously described (25–30, 33, 34, 42). The UM-PDX-HACC-14 model was generated in our laboratory from a metastatic adenoid cystic carcinoma, as described (42). Positive staining for HLA isolated human cells and were incubated for 10″ at 4°C. The rationale for our first experiment was to inject serially diluted ALDH^high^CD44^high^ cells, as described (22, 25). Serial dilution experiments were performed by decreasing the number of cells by a factor of 10, starting from 1,500 ALDH^high^CD44^high^ cells. As controls, we injected 10-fold more ALDH^low^CD44^low^ cells than the highest number of ALDH^high^CD44^high^ cells (i.e., 15,000). Cells were injected directly into the mouse submandibular glands (*n* = 2/group) with a follow-up of 13 months (41, 42). In a second experiment designed to verify our initial *in vivo* results, we injected 40,000 or 4,000 ALDH^high^CD44^high^ or pooled cells (i.e., ALDH^low^CD44^low^ + ALDH^low^CD44^high^ + ALDH^high^CD44^low^) and followed mice (*n* = 3/group) for up for 16 months. Autopsies were performed in all mice to examine for presence of metastases. All *in vivo* work was performed under an approved protocol according to the University of Michigan Animal Care and Use Committee (IACUC) guidelines. Animals were anesthetized during surgical procedures and received post-operatory analgesics to minimize discomfort or pain.

### Immunohistochemistry

All mouse salivary and lung tissues were stained with Hematoxylin and Eosin (H and E) and evaluated by two experienced oral pathologists (FN, RC). ALDH1A1 (1:1,000, Santa Cruz, SC-374149) and CD44 expression (1:50, Cell Signaling, # 3570) staining of tumors was performed as previously described (42). Human tumors were confirmed by positive anti-human HLA class 1 ABC (1:25 EMR8-5; Abcam, Cambridge, UK) or Keratin-7 (1:50; Cell Signaling) staining and an absence of anti-mouse CD-45 (1:100 clone 30-F11; Biolegend, San Diego, CA, USA), a marker of mouse inflammatory cells (42).

### Salisphere assays

Primary spheres were generated from unsorted or sorted UM-HACC cells (ALDH^low^CD44^low^, ALDH^low^CD44^high^, ALDH^high^CD44^low^ or ALDH^high^CD44^high^) and grown in ultra-low attachment (ULA) conditions (25–30, 34). *In vitro* serial dilution studies used UM-HACC-2A (4,000, 400, 40 and 4) or UM-HACC-14 (10,000, 1,000, 100, 10) cells/well in 6-well ULA plates in triplicate and spheres were counted every 1–3 days (25–30, 34). To generate secondary spheres, sorted ALDH^high^CD44^high^ or ALDH^low^CD44^low^ (5.0 × 10^5^–1 × 10^6^) cells were cultured in T-75 ULA flasks for 4–7 days. Primary spheres were collected in a 40 µM cell strainer, washed in PBS, dissociated into single cells (FACSMAX, Genlantis, San Diego, CA) counted and re-plated into 6-well ULA plates for 7 days (25–30, 34). Salispheres were cultured in DMEM/F-12 (Invitrogen) supplemented with 20 ng/ml EGF (Sigma-Aldrich), 20 ng/ml basic fibroblast growth factor (bFGF; Millipore, Burlington, MA), 1% penicillin/streptomycin (Invitrogen), 1% glutamax (Invitrogen), 1% N-2 supplement (Invitrogen), 1 μM dexamethasone (Sigma-Aldrich), and 10 μg/ml insulin (Sigma-Aldrich) (25–30, 34).

### Statistical analysis

All statistical analyses were performed using the Prism software (GraphPad; San Diego, CA). Comparisons between 2 experimental conditions were analyzed by paired *t*-tests, while one-way ANOVA followed by *post-hoc* tests was utilized for multiple group analyses. Statistical significance was determined at *p* < 0.05.

## Results

### Characterization of ALDH and CD44 in human salivary gland ACC cell lines

**Figure 1:**
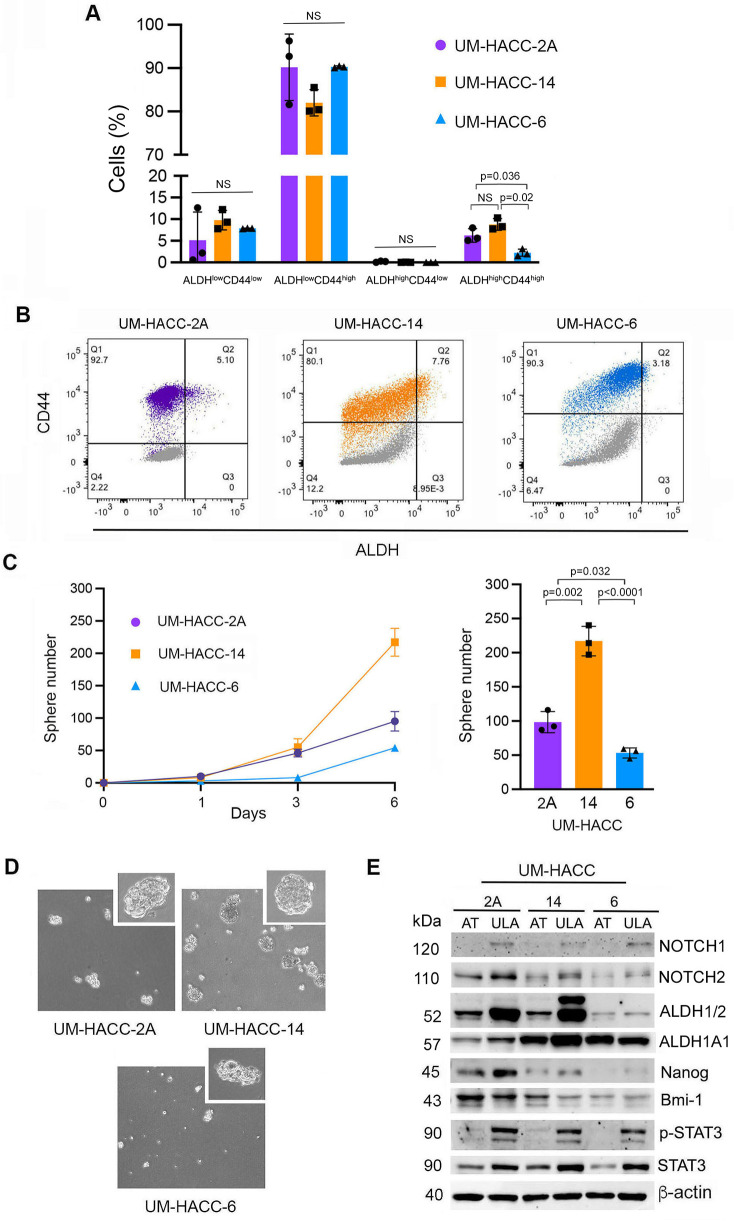
Baseline characterization of cancer stem cell fraction, sphere number and protein expression in UM-HACC cell lines. **(A)** Bar graph showing the cancer stem cell (CSC) and non-CSC percentages in flow cytometry sorted cells. **(B)** Representative flow cytometry gating schematics **(C)**. Line and bar graphs depicting the average sphere number in unsorted UM-HACC cell lines. **(D)** Photomicrographs of spheres. **(E)** Western blots of UM-HACC cells grown in attached (AT) or ultra-low attachment (ULA) conditions. Statistical significance was defined at *p* < 0.05, as determined by one-way ANOVA followed by *post-hoc* analyses. NS, non-significant.

To begin to understand the pattern of expression of ALDH and CD44 in ACC, three human salivary gland ACC cell lines were evaluated using flow cytometry. The average percentage of CSC (ALDH^high^CD44^high^) was approximately 5%, 7% and 3% in UM-HACC-2A, UM-HACC-14 and UM-HACC-6 cells respectively (Figure 1A). The gating strategy used to isolate CSC is shown in (Figure 1B). We observed that the proportion of ALDH^low^CD44^high^ was the highest (80%–90%), ALDH^high^CD44^low^ was minimal, and the percentage of ALDH^low^CD44^low^ ranged from 5% to 12% (Figure 1A). There were significantly higher levels of CSC (ALDH^high^CD44^high^) in UM-HACC-2A and UM-HACC-14 cells when compared to UM-HACC-6 (*p* < 0.05). There were no significant differences in the non-CSC fraction (ALDH^high^CD44^low^, ALDH^low^CD44^high^, ALDH^low^CD44^low^) for each UM-HACC line (*p* > 0.05). Each cell line was analyzed in triplicate experiments with reproducible results.

To determine if UM-HACC cell lines generate salispheres, 5,000 cells were cultured in ultra-low attachment plates. UM-HACC-14 cells formed the highest average number of salispheres when compared to UM-HACC-2A and UM-HACC-6 cells after 6 days (Figure 1C). Photomicrographs showed well-defined salispheres for all ACC cell lines (Figure 1D). Western blotting revealed an overall trend for enhanced stemness markers in ACC cells suspended in ultra-low attachment plates, as expected. We observed increased expression of Notch1, Notch2, and Nanog in salispheres compared to attached cells (Figure 1E). ALDH1A1 and ALDH1/2 were increased in UM-HACC-2A and UM-HACC-14 salispheres when compared to attached cells, while UM-HACC-6 salispheres had similar expression levels. Interestingly, we observed an additional, higher molecular weight band in UM-HACC-14 salispheres in the Western blot for ALDH1/2. Both constitutive p-STAT3 and total STAT3 expression were also upregulated in salispheres when compared to attached cells in the ACC cell lines evaluated here (Figure 1E). And finally, Bmi-1 levels were similar when attached cells were compared to suspended cells in our cell lines (Figure 1E).

### ALDH^high^CD44^high^ ACC cells self-renew and show strong expression of stem cell markers

**Figure 2:**
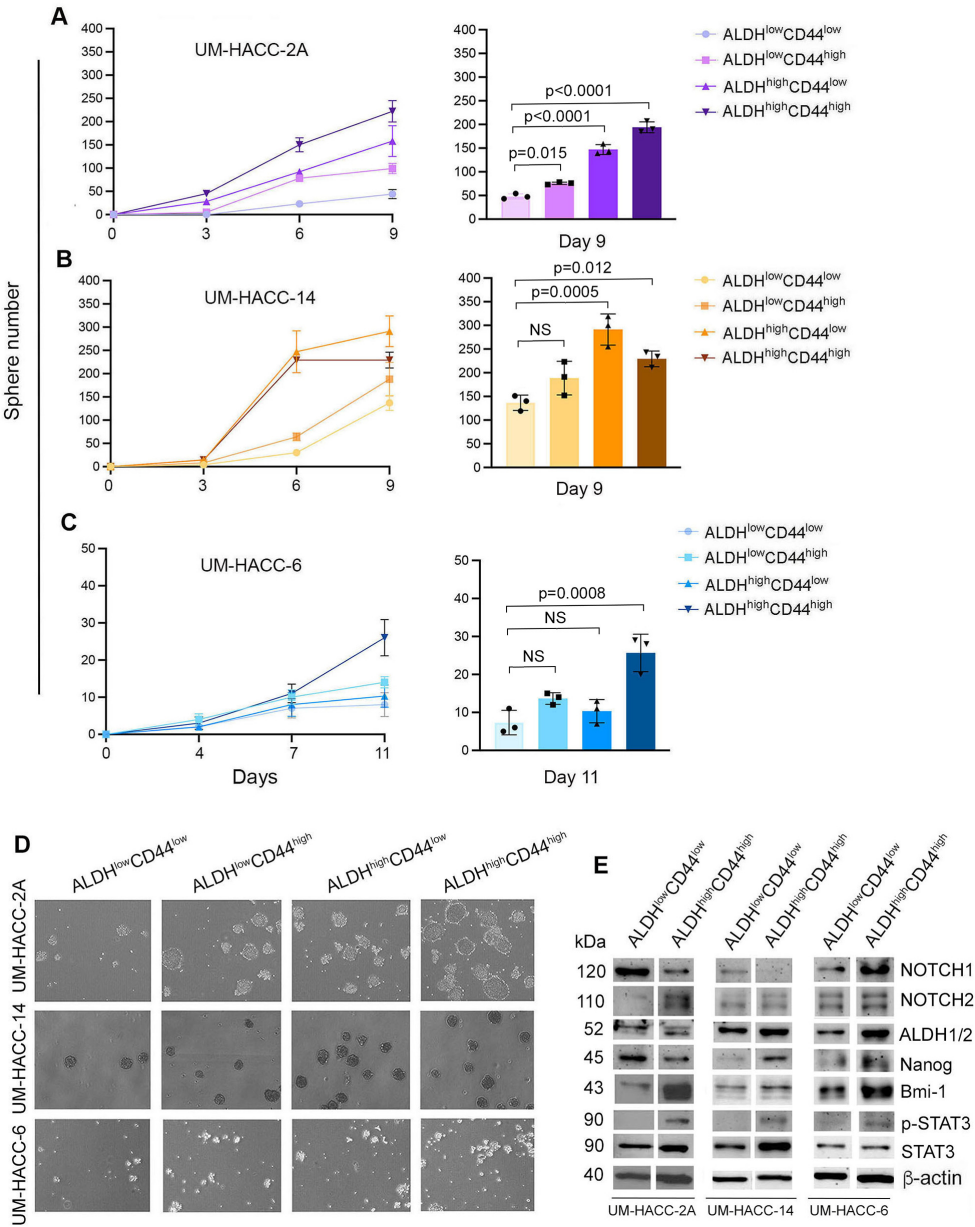
ALDH^high^CD44^high^ cells form more spheres and express higher levels of ALDH1-2, NOTCH2, p-STAT3 and Bmi-1 compared to non-CSC. **(A–C)** Line and bar graphs showing the number of spheres in UM-HACC-2A, UM-HACC-14 and UM-HACC-6 cell lines. Statistical significance was defined at *p* < 0.05, as determined by paired *t*-tests using ALDH^low^CD44^low^ cells as controls. NS, non-significant. **(D)** Photomicrographs of spheres generated from ALDH^high^CD44^high^ CSC and non-CSC (ALDH^low^CD44^low^, ALDH^low^CD44^high^, ALDH^high^CD44^low^). **(E)** Western blot analysis in ALDH^high^CD44^high^ and ALDH^low^CD44^low^ cells.

To determine if ALDH^high^CD44^high^ cells had enhanced *in vitro* stemness features, UM-HACC-2A, UM-HACC-14 and UM-HACC-6 cells were sorted for all four marker combinations (i.e., ALDH^low^CD44^low^, ALDH^low^CD44^high^, ALDH^high^CD44^low^, ALDH^high^CD44^high^) and then plated in ultra-low attachment conditions immediately after sorting (Figures 2A–C). The flow cytometry gating strategy for *in vitro* experiments is shown in (Supplementary Figure A1). We observed that ALDH^high^CD44^high^ cells formed the highest average salisphere number in UM-HACC-2A and UM-HACC-6 cells compared to all other groups (Figures 2A–C). UM-HACC-14 behaved slightly differently. In this case, ALDH^high^CD44^high^ and ALDH^high^CD44^low^ cells formed significantly more salispheres compared to ALDH^low^CD44^low^ cells but were not statistically different from each other (Figure 2B). Photomicrographs showed a trend for increased numbers of salispheres in ACC cells expressing high levels of ALDH activity (Figure 2D). We performed western analysis of several markers of stemness (i.e., Notch1, Notch2, ALDH1A1, ALDH1/2, Nanog, Bmi-1) in the 3 cell lines immediately after sorting for ALDH activity and CD44 expression. UM-HACC-2A and UM-HACC-14 sorted cells had similar protein expression profiles, i.e., Notch2, ALDH1/2, Bmi-1, p-STAT3, and STAT3 were increased in ALDH^high^CD44^high^ cells compared to ALDH^low^CD44^low^ cells (Figure 2E). Notch1 expression was reduced ALDH^high^CD44^high^ cells in UM-HACC-2A and UM-HACC-14, and upregulated in ALDH^high^CD44^high^ cells in the UM-HACC-6 cell line. Nanog expression was reduced in UM-HACC-2A ALDH^high^CD44^high^ cells and increased in UM-HACC-14 and UM-HACC-6 ALDH^high^CD44^high^ cells, when compared to ALDH^low^CD44^low^ cells. Interestingly, ALDH^high^CD44^high^ cells sorted from UM-HACC-6 cell line showed increased expression of all CSC markers evaluated here (i.e., Notch1, Notch2, ALDH1/2, Nanog, Bmi-1) when compared to ALDH^low^CD44^low^ cells (Figure 2E).

**Figure 3:**
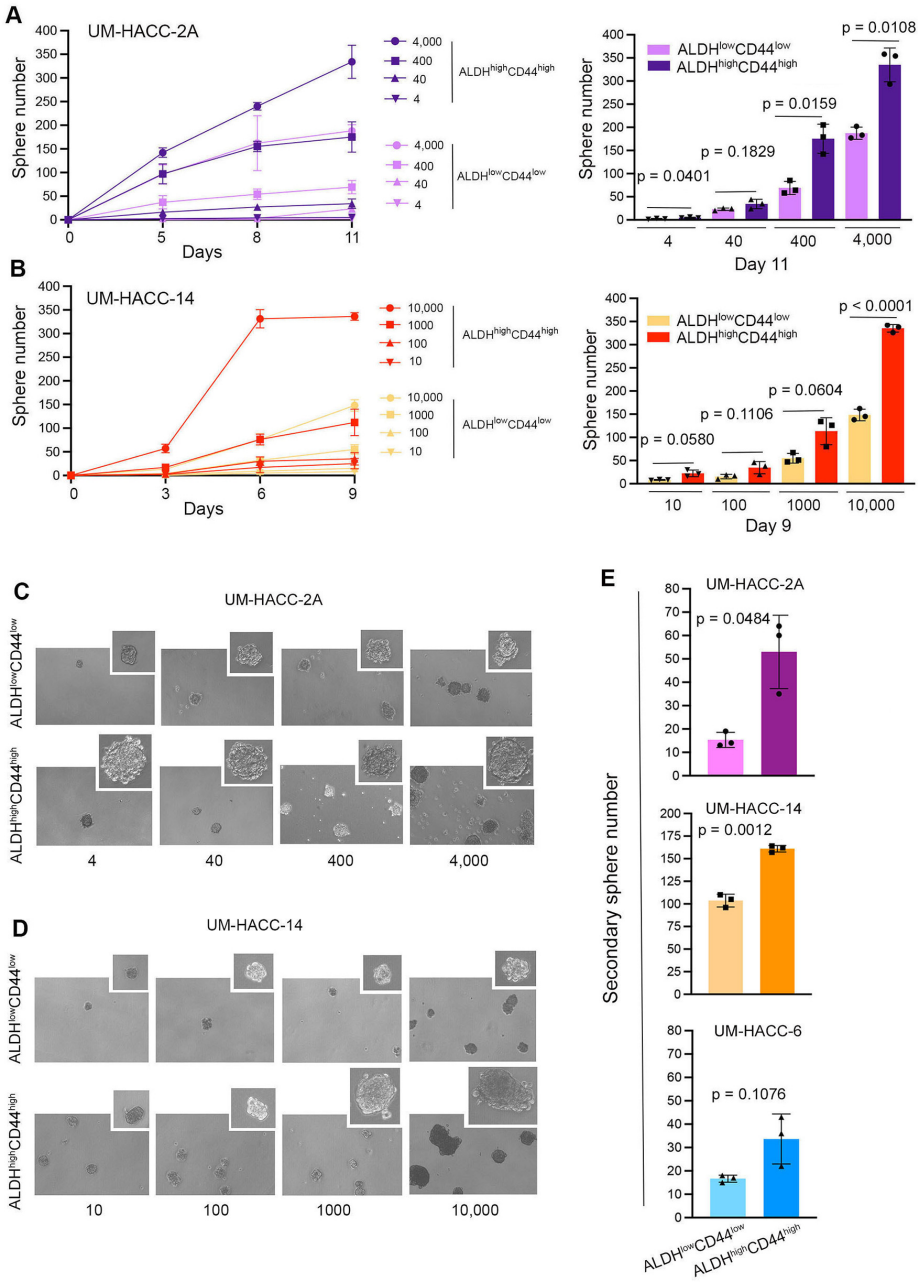
ALDH^high^CD44^high^ cells form more spheres and exhibit enhanced self-renewal capabilities compared to ALDH^low^CD44^low^ cells. **(A,B)** Line and bar graphs showing sphere number after sorting and serial dilution of UM-HACC-2A and UM-HACC-14 cells. *p* < 0.05. **(C,D)** Photomicrographs of spheres generated from ALDH^high^CD44^high^ and ALDH^low^CD44^low^ cells. **(E)** Bar graphs displaying the average number of secondary spheres generated with UM-HACC cell lines after 7 days.

To verify our initial results, we performed an independent serial dilution experiment *in vitro* with UM-HACC-2A and UM-HACC-14 cells sorted for ALDH^high^CD44^high^ and ALDH^low^CD44^low^ (Figures 3A,B). Here, we observed similar overall trends as those showed in the previous experiments, with the most noticeable differences observed at the higher numbers of cells plated. Interestingly, the salispheres generated by ALDH^high^CD44^high^ cells tended to be larger than those formed with ALDH^low^CD44^low^ cells (Figure 3C). To understand the impact of ALDH activity and CD44 expression on self-renewal of ACC cells, we sorted the 3 cell lines for ALDH^high^CD44^high^ or ALDH^low^CD44^low^ and expanded them as primary salispheres for 7 days. Then, we generated secondary spheres by dissociation of the primary salispheres, counting and re-plating the cells into new ultra-low attachment plates. The ALDH^high^CD44^high^ cells formed consistently more secondary salispheres than the ALDH^low^CD44^low^ cells in the ACC cell lines evaluated here (Figure 3E).

### ALDH^high^CD44^high^ ACC cells are highly tumorigenic and generate lung metastases

**Figure 4:**
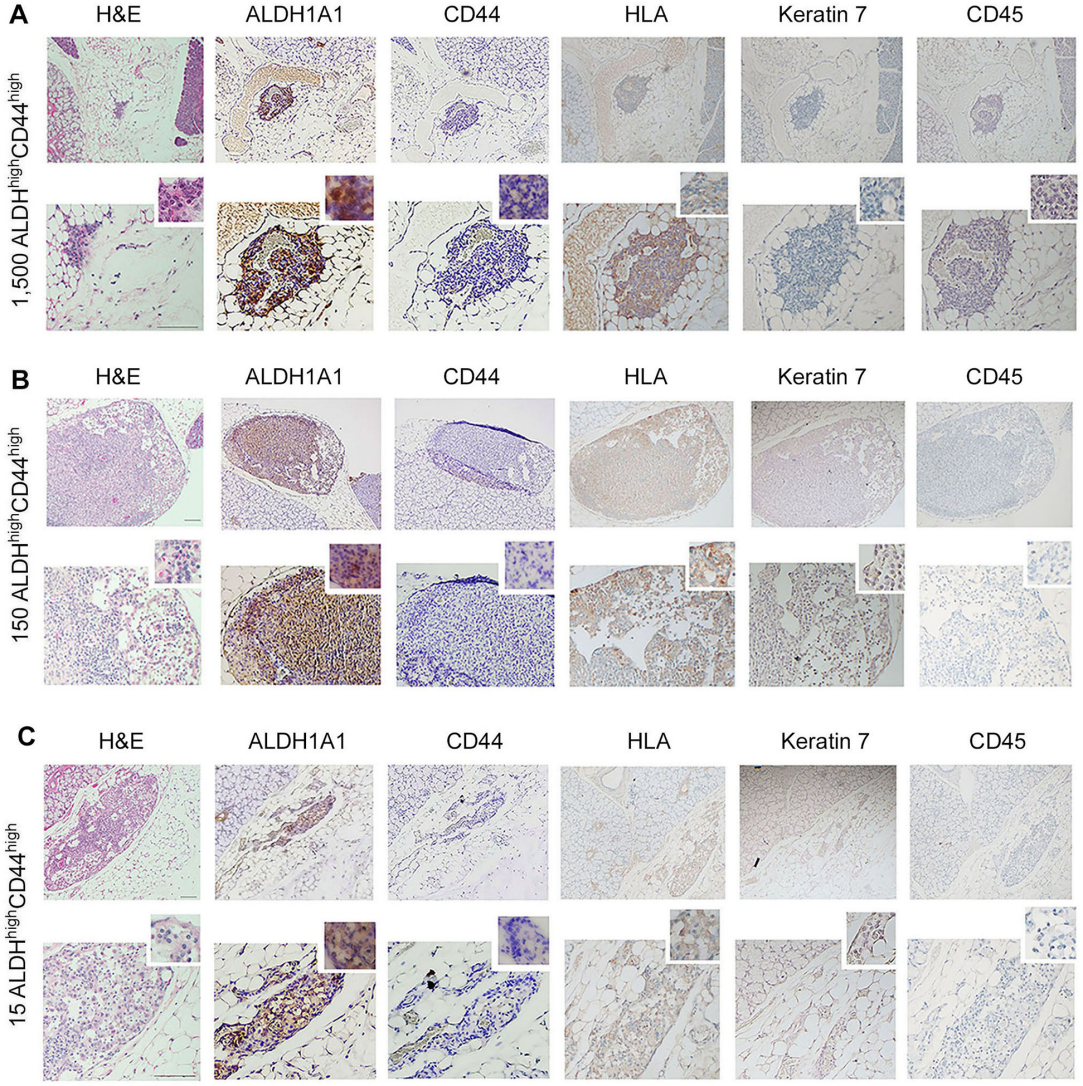
Tumorigenicity of UM-PDX-HACC-14 cancer stem cells in mouse salivary gland. **(A-C)** Photomicrographs of orthotopic tumors generated with 1,500, 150 and 15 UM-PDX-HACC-14 ALDH^high^CD44^high^ cells and stained with Hematoxylin/Eosin (H&E) or immunohistochemistry with anti-human ALDH1A1, CD44, HLA antibody, anti-Keratin 7 antibody and with anti-mouse CD45 antibody. All images are shown at 40× (top rows) and 100× magnification (bottom rows) with a 200× magnification shown in the smaller window. Scale bar represents 100 µm.

**Figure 5:**
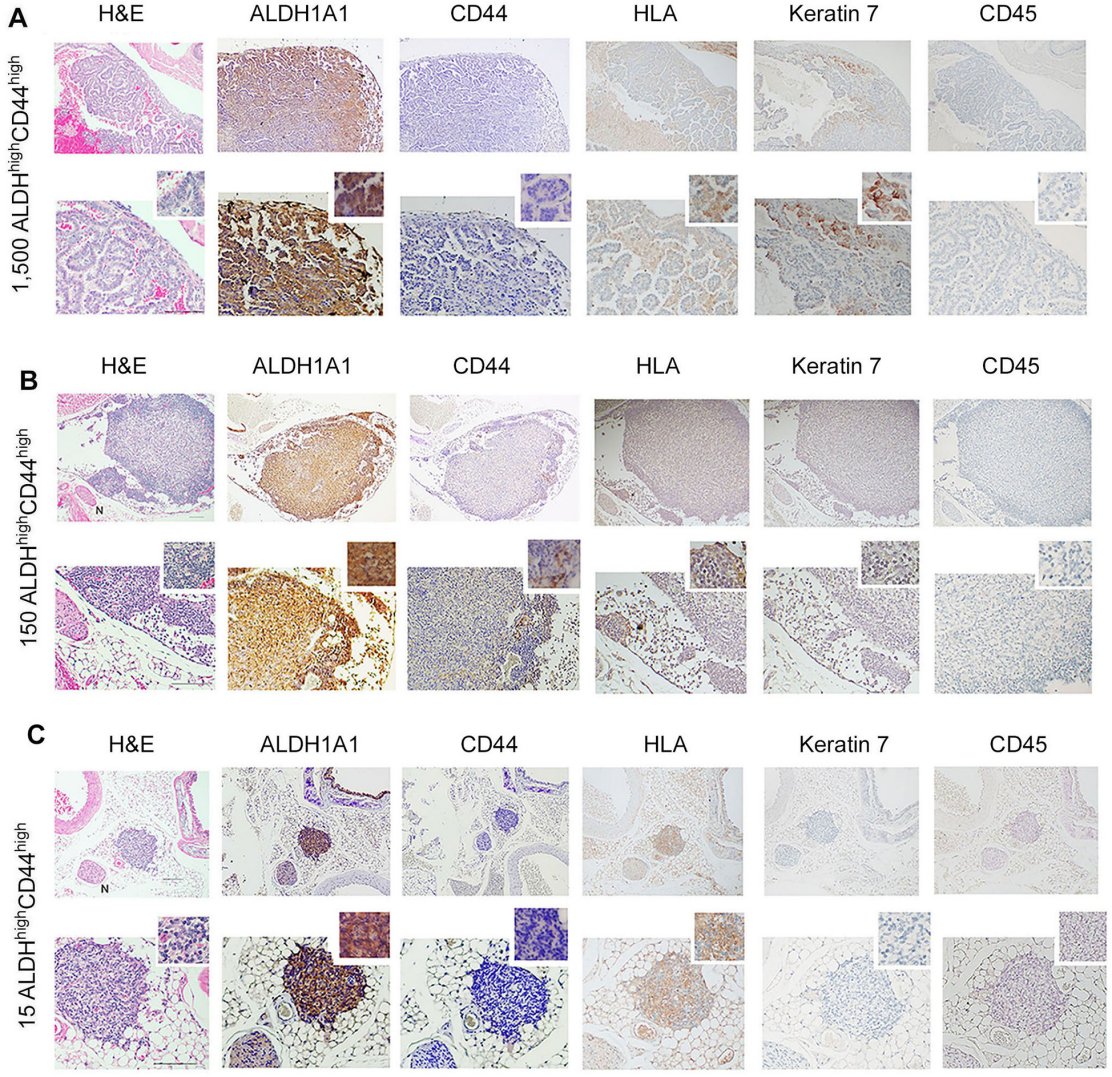
Metastatic capability of UM-PDX-HACC-14 cancer stem cells. **(A-C)** Photomicrographs of lung metastasis generated by orthotopic injection of 1,500, 150 and 15 UM-PDX-HACC-14 ALDH^high^CD44^high^ cells and stained with Hematoxylin/Eosin (H&E), or immunohistochemistry with anti-human ALDH1A1, CD44, HLA antibody, anti-Keratin 7 antibody and with anti-mouse CD45 antibody. N indicates a nerve in the H&E panel, 40× view **(B,C)**. All images are shown at 40× (top rows) and 100× magnification (bottom rows) with a 200× magnification shown in the smaller window. Scale bar represents 100 µm.

To understand whether our *in vitro* findings were consistent with the behavior of cells *in vivo*, we performed two independent serial dilution experiments involving the sorting of the ACC cells and transplantation into mice. Here, we grew ACC PDX tumors in mice (UM-PDX-HACC-14), retrieved them and dissociated the tumors to prepare single-cell suspensions, and then flow sorted these cells for ALDH activity and CD44 expression. The flow cytometry gating strategies used for the *in vivo* study is shown in Supplementary Figure 1B. These cells were injected into the submandibular salivary glands of mice immediately after sorting. Hematoxylin and Eosin staining of the mouse salivary glands showed human tumor cells (i.e., HLA-positive cells) upon injection of very low numbers (15–1,500) ALDH^high^CD44^high^ cells (Figures 4A–C). Strong expression of ALDH1A1 and minimal CD44 staining was present in salivary gland tumors generated with ALDH^high^CD44^high^ cells (Figures 4A–C). No staining of mouse CD45 confirmed that the cells growing tumors in the salivary glands were of human origin. As expected, there was minimal staining for Keratin-7, a marker used to detect differentiated tumor cells (42). These tumors grew very slowly and did not show palpability even after a 13-month follow-up, which is consistent with the slow growth rate observed in patients with ACC. Surprisingly, despite very small primary tumors, we observed that a significant number of mice exhibited lung metastases. We observed several lung metastases that stained positive with HLA and keratin 7 and showed no staining with mouse CD-45 (Figures 5A–C). Similar to the primary tumors in the salivary glands, strong ALDH1A1 staining and minimal CD44 expression was detected in the lung metastases (Figures 5A–C). Notably, some of these metastatic sites were localized in close proximity to nerves (Figures 5B,C).

**Figure 6:**
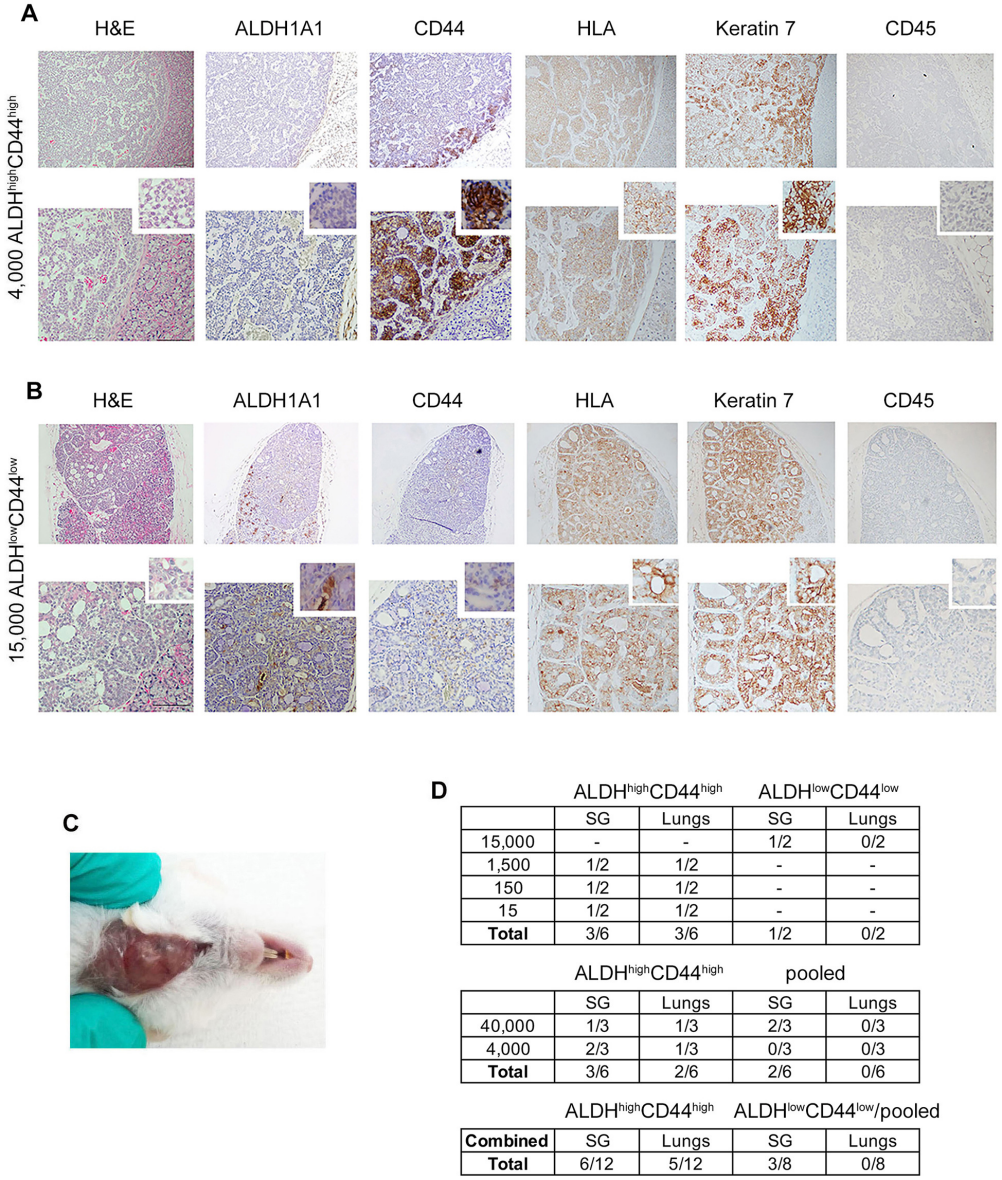
Different ACC tumor phenotypes generated with UM-PDX-HACC-14 cancer and non-cancer stem cells in mouse submandibular gland. **(A)** Photomicrographs of an orthotopic solid salivary gland tumor after injection of 4,000 ALDH^high^CD44^high^ (7 months). **(B)** Photomicrograph of an orthotopic cribriform/tubular salivary gland tumor after injection of 15,000 ALDH^low^CD44^low^ cells (13 months) stained with Hematoxylin/Eosin (H&E), or immunohistochemistry with anti-human ALDH1A1, CD44, HLA antibody, anti-Keratin 7 antibody and with anti-mouse CD45 antibody. All images are shown at 40× (top rows) and 100× magnification (bottom rows) with a 200× magnification shown in the smaller window. Scale bar represents 100 µm. **(C)** Photograph of the solid palpable salivary gland tumor of UM-PDX-HACC-14 ALDH^high^CD44^high^ cells in **(A,D)** table depicting the total number of mouse salivary gland and lung tumors after orthotopic injection of ALDH^high^CD44^high^, ALDH^low^CD44^low^ or pooled cells (ALDH^low^CD44^low^, ALDH^low^CD44^high^, ALDH^high^CD44^low^).

To further explore the tumorigenic potential of ALDH^high^CD44^high^ ACC cells, we evaluated their tumorigenic potential against pooled cells (ALDH^low^CD44^low^, ALDH^low^C44^high^, ALDH^high^CD44^low^) for 16 months in mice (Figures 6A,B). Hematoxylin and eosin staining revealed a salivary gland tumor with poorly differentiated, solid phenotype with low ALDH1A1 and strong CD44, HLA and keratin-7 expression (Figure 6A). In contrast, the injection of 15,000 ALDH^low^CD44^low^ cells resulted in a well-differentiated salivary gland tumor 13 months after injection (Figure 6B). Hematoxylin and eosin staining confirmed cribriform/tubular morphology, positive staining for HLA and keratin-7 and no CD45 staining (Figure 6B). We observed a palpable tumor 7 months after orthotopic injection of 4,000 ALDH^high^CD44^high^ cells (Figure 6C). When we combined both *in vivo* experiments together, a total of 12 mice injected with ALDH^high^CD44^high^ ACC cells resulted in 6 primary salivary gland tumors (50%) and 5 lung metastases (42%). In contrast, injection of 8 mice with control cells (i.e., ALDH^low^CD44^low^ or pooled cells) gave rise to 3 primary salivary gland tumors (37%) and 0 lung metastases (0%) (Figure 6D).

## Discussion

The lack of better mechanistic understanding of the pathobiology of ACC has contributed to the poor long-term clinical outcome of patients with ACC. One of the key features of ACC is tumor cell heterogeneity, with tumors presenting in a cribriform or tubular pattern, while more undifferentiated tumors typically present a solid pattern. Whether or not ACC have uniquely tumorigenic cells and how these cells can be identified remains unclear. Here, we have shown that ALDH^high^CD44^high^ cells are more tumorigenic than controls. Further, we showed that orthotopic tumors generated by ALDH^high^CD44^high^ cells have a higher potential for metastatic spread to the lungs when compared with control cells. We report here that ALDH^high^CD44^high^ ACC cells generated more orthotopic tumors than ALDH^low^CD44^low^ cells, which is consistent with data published by Keysar and colleagues (36). We have also reported that ALDH^high^CD44^high^ ACC cells are more prone to metastatic spread to the lungs than ALDH^low^CD44^low^ cells.

Two recent studies have reported positive tumor cell staining for ALDH and CD44 in malignant salivary gland tumors (43, 44). Santos and colleagues reported variable ALDH expression in ACC tumors and consistent ALDH1 stromal cell expression correlated with reduced overall disease-free survival and advanced staging (44). We report here strong ALDH1A1 staining and lower CD44 staining in most tumors generated with ALDH^high^CD44^high^ cells. Similar trends were observed in metastatic lung tumors. Interestingly, even when we implanted a very low number of ALDH^high^CD44^high^ cells (15 cells) into the mouse submandibular gland, we observed metastatic spread to the lungs. While these correlational *in vivo* results suggest that ALDH1A1 (not CD44) is the primary “driver” of ACC tumorigenesis, additional mechanistic and *in vivo* studies should be done to verify this hypothesis. Considering that prevention and treatment of metastases require systemic therapies, these findings provide evidence in support of strategies for therapeutic targeting of ACC cancer stem cells (i.e., ALDH^high^CD44^high^cells).

Studies have shown that several ALDH isoforms play a role in the pathobiology of cancer and in resistance to anti-cancer therapy (45). We observed here that the histological features of the tumors generated with orthotopic injection of ALDH^high^CD44^high^ cells matched the solid pattern exhibited by the patient that donated the tissues for the generation of the PDX model used here, UM-PDX-HACC-14 (42). The overall number of tumors generated with ALDH^low^CD44^low^ cells was smaller and their histological features were different than those observed in the tumors generated by the ALDH^high^CD44^high^ cells. The tumors generated with ALDH^high^CD44^high^ cells were of a solid, very aggressive phenotype, while the tumors generated with control cells presented with a less aggressive, cribriform/tubular histology. The connection of CSC to ACC histology sub-types was previously alluded to using immunohistochemistry for CD133 and CD44 in histological sections of 26 human ACC tumors (46). The authors showed that CD133 + and CD44 + cells lined cribriform pseudocysts and accumulated in tubular ACC. The results of this correlation study were confirmed by our results, which showed that CSCs defined by ALDH activity and CD44 expression generate solid, more aggressive ACC tumors while control cells generated a cribriform/tubular, more differentiated tumor. A limitation of our study is the small number ACC cell lines and animal models available for mechanistic studies. Nevertheless, we believe that the data presented here provide preliminary support for further exploration of the function of cancer stem cells in salivary gland adenoid cystic carcinoma.

The tumor progression observed in our experiments mimics the features of ACC tumors in patients (1–3, 6). While transplantation of HNSCC ALDH^high^CD44^high^ cells generates tumors within 4–6 months (22), we observed here that generation of orthotopic ACC tumors and lung metastases typically requires 13–16 months, which is approximately half of the life span of a mouse. This result is consistent with the slow, progressive disease and frequent lung metastases observed in patients with ACC. Notably, we unveiled here the generation of salivary gland ACC and lung metastases with very low ACC CSC cell numbers (as low as 15 cells). On the other hand, we have not observed bone or liver metastases in our studies. Another interesting feature is that we observed proximity between nerves and ACC cells in lung metastatic sites. This feature suggests that the orthotopic model reproduces, at least in part, the perineural invasion presented by the patient ACC that was used to generate our PDX model. Of note, perineural invasion is common feature of patients with ACC (1–3, 6, 42).

Progress in identifying and understanding the role of CSC in adenoid cystic carcinoma has been slow due to the lack of available authentic, non-transformed cell lines. Phuchareon and colleagues identified the cross contamination of 6 ACC cell lines widely used to study this disease (47). Here we report stemness of ACC using salisphere assays and analysis of cancer stem cell markers *in vitro* with our 3 UM-HACC cell lines and one matching PDX model (41, 42). The enhanced expression of Notch1, Notch2, and Nanog in salispheres and ALDH^high^CD44^high^ cells suggests the function of cancer stem cells in ACC. Of note, increased Notch1 and Notch2 in the 3 cell lines is particularly exciting, since they are therapeutic targets being studied in patients with ACC (48). We observed an extra band with slightly higher molecular weight in the western blot from UM-HACC-14 salispheres. The postulate that this extra band is likely due to enhanced expression of ALDH2 (56 kDa) in addition to ALDH1 (55 kDa). Of note, the antibody used here recognizes both, ALDH1 and ALDH2 isoforms. The extra band could also be due to post translational modification of ALDH1. For example, glycosylation events have been described for this molecule (45, 49).

When we sorted cancer stem cells (ALDH^high^CD44^high^) from UM-HACC-6, we observed increased expression of CSC markers despite the fact this cell line has the lowest percentage of CSC and the slowest salisphere formation. This cell line is derived from an aggressive, recurrent ACC tumor with signs of perineural invasion and presence of lung metastases (32, 42). Orthotopic injection of UM-HACC-6 cells into mouse submandibular glands resulted in lung metastases within 6 months (42). We are currently performing additional studies to better understand the unique aspects of the stemness and tumorigenic potential of this ACC cell line.

The difficulty of diagnosing and treating ACC is related to the rarity of this tumor and the heterogeneity of tumor presentation. ACC tumor size, grade, stage, +/− metastases, (lymph or hematic spread), +/− perineural invasion, and fusion status makes predictions of rate of tumor progression and long-term prognosis very difficult. We have spent the last 15 years generating ACC cell lines. The UM-HACC-2A is c-MYB-NFIB positive cell line (32, 41, 42). The UM-HACC-14 cell line and UM-HACC-6 cell lines are fusion-negative (42). Despite differences in fusion status, tumor origin, location and grade, these three human ACC cell lines exhibit multipotent and self-renewing cancer stem cells with uniquely high tumorigenic potential. These findings suggest that cancer stem cells might be considered a common treatment target shared by diverse ACC tumor phenotypes. As such, the work presented here contributes to the knowledge of the pathobiology of this rare malignancy and suggest a new cellular target that can be explored to develop novel therapeutic strategies for patients with salivary gland adenoid cystic carcinoma.

## Data Availability

The datasets presented in this article are not readily available because there are no data sets associated with this work. Requests to access the datasets should be directed to not applicable.

## Supplementary material

Supplementary Figure 1 **(A)** Representative flow plots and gating strategy for *in vitro* sorting of UM-HACC-2A for ALDH activity and CD44 expression. **(B)** Representative flow plots and gating strategy for sorting of UM-PDX-HACC-14 cells retrieved from mice for ALDH activity and CD44 expression.
