# Supplementary figures and images for: Characterization of uniquely tumorigenic cancer stem cells in salivary gland adenoid cystic carcinoma
Source: Front Oral Health. 2025 Apr 30;6:1570042. doi: 10.3389/froh.2025.1570042 (PMC12075560; doi:10.3389/froh.2025.1570042)

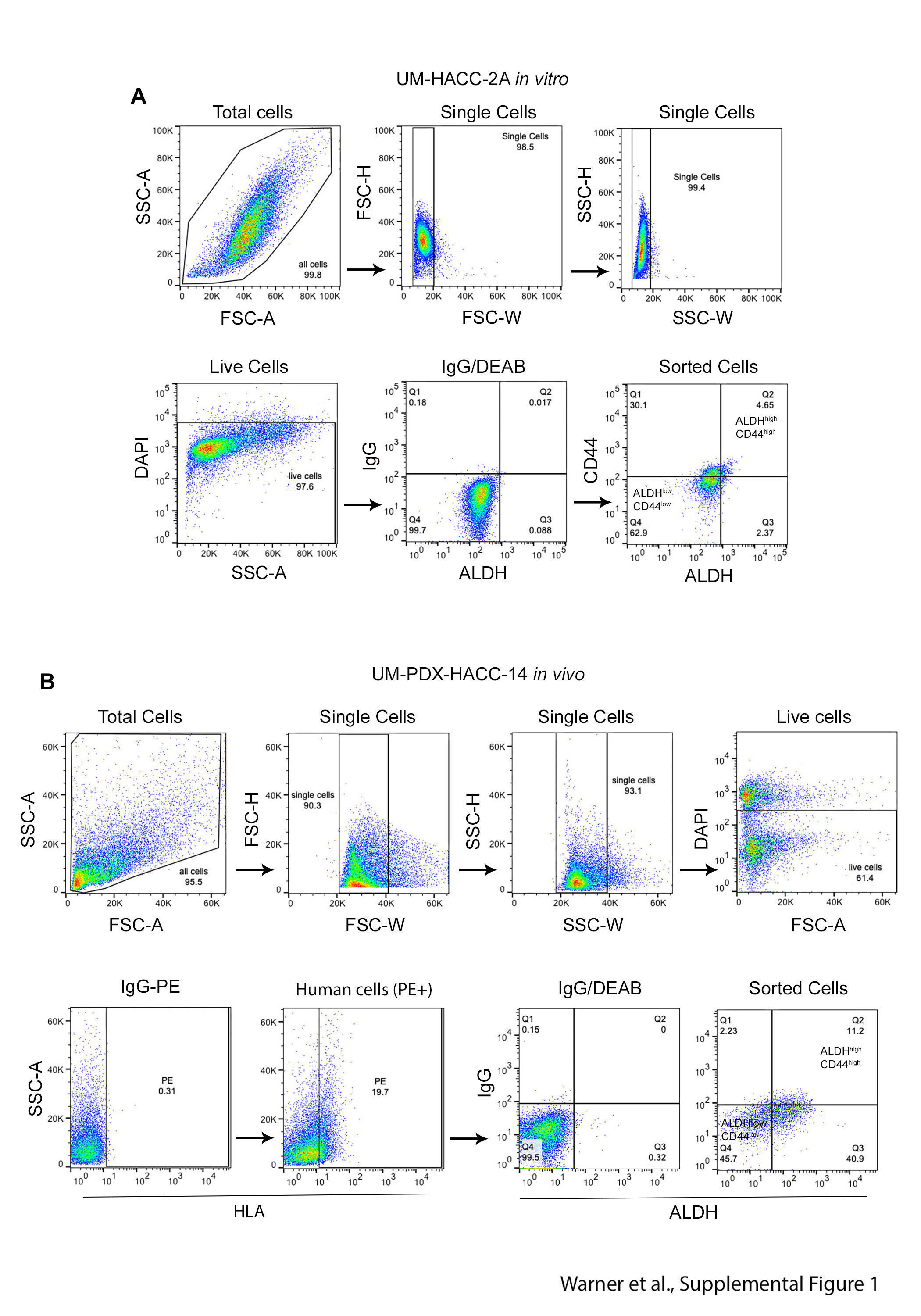

Supplement: Supplementary Figure 1 — (A) Representative flow plots and gating strategy for in vitro sorting of UM-HACC-2A for ALDH activity and CD44 expression. (B) Representative flow plots and gating strategy for sorting of UM-PDX-HACC-14 cells retrieved from mice for ALDH activity and CD44 expression. [file Image1.jpeg]
